# Genetic diversity, extent of linkage disequilibrium and persistence of gametic phase in Canadian pigs

**DOI:** 10.1186/s12863-017-0473-y

**Published:** 2017-01-21

**Authors:** Daniela A. Grossi, Mohsen Jafarikia, Luiz F. Brito, Marcos E. Buzanskas, Mehdi Sargolzaei, Flávio S. Schenkel

**Affiliations:** 1 Centre for Genetic Improvement of Livestock, University of Guelph, Guelph, Ontario Canada; 2 Canadian Centre for Swine Improvement Inc, Ottawa, Ontario Canada; 3 Departamento de Zootecnia, Centro de Ciências Agrárias - Campus II, Universidade Federal da Paraíba, Areia, Paraíba Brazil; 4 The Semex Alliance, Guelph, Ontario Canada

**Keywords:** Effective population size, Linkage disequilibrium, Pig breeds, Population structure, Runs of homozygosity

## Abstract

**Background:**

Knowledge on the levels of linkage disequilibrium (LD) across the genome, persistence of gametic phase between breed pairs, genetic diversity and population structure are important parameters for the successful implementation of genomic selection. Therefore, the objectives of this study were to investigate these parameters in order to assess the feasibility of a multi-herd and multi-breed training population for genomic selection in important purebred and crossbred pig populations in Canada. A total of 3,057 animals, representative of the national populations, were genotyped with the Illumina Porcine SNP60 BeadChip (62,163 markers).

**Results:**

The overall LD (*r* ^2^) between adjacent SNPs was 0.49, 0.38, 0.40 and 0.31 for Duroc, Landrace, Yorkshire and Crossbred (Landrace x Yorkshire) populations, respectively. The highest correlation of phase (r) across breeds was observed between Crossbred animals and either Landrace or Yorkshire breeds, in which r was approximately 0.80 at 1 Mbp of distance. Landrace and Yorkshire breeds presented *r* ≥ 0.80 in distances up to 0.1 Mbp, while Duroc breed showed *r* ≥ 0.80 for distances up to 0.03 Mbp with all other populations. The persistence of phase across herds were strong for all breeds, with *r* ≥ 0.80 up to 1.81 Mbp for Yorkshire, 1.20 Mbp for Duroc, and 0.70 Mbp for Landrace. The first two principal components clearly discriminate all the breeds. Similar levels of genetic diversity were observed among all breed groups. The current effective population size was equal to 75 for Duroc and 92 for both Landrace and Yorkshire.

**Conclusions:**

An overview of population structure, LD decay, demographic history and inbreeding of important pig breeds in Canada was presented. The rate of LD decay for the three Canadian pig breeds indicates that genomic selection can be successfully implemented within breeds with the current 60 K SNP panel. The use of a multi-breed training population involving Landrace and Yorkshire to estimate the genomic breeding values of crossbred animals (Landrace × Yorkshire) should be further evaluated. The lower correlation of phase at short distances between Duroc and the other breeds indicates that a denser panel may be required for the use of a multi-breed training population including Duroc.

**Electronic supplementary material:**

The online version of this article (doi:10.1186/s12863-017-0473-y) contains supplementary material, which is available to authorized users.

## Background

The continued growth in the world human population has been accompanied by a larger demand for animal products, such as meat. Worldwide, pork is the most heavily consumed meat, especially in America, Europe and Asia. It accounts for 36.3% of production, followed by poultry (34.4%) and beef (21.2%) [1]. Pork consumers are demanding animals that are raised under exemplary welfare conditions and produce tasty meat in a cost-effective manner. In order to achieve these requirements, pig breeders have improved environmental and welfare conditions and heavily invested in genetic selection to increase genetic progress for desirable traits and consequently, the industry profitability. Despite the genetic progress achieved through traditional genetic evaluations, advances in the area of genomics and genomic technologies have created great opportunities to increase the rate of genetic gain per year, through genomic selection (GS, [2]). Genomic selection has been successfully implemented in dairy cattle [3, 4] and is under development or in implementation stage in many other livestock species [5–10].

Currently, two SNP panels have become commercially available for pigs: the Illumina Porcine SNP60 BeadChip and the GeneSeek Genomic Profiler for Porcine high-density BeadChip, containing approximately 60 and 70 thousand single nucleotide polymorphisms (SNPs), respectively. The availability of such tools enhanced research on genomics. For example, the pig Quantitative Trait Loci (QTL) database (http://www.animalgenome.org) contains more than 15,000 QTLs for health, production, reproduction, as well as meat and carcass quality traits. QTL identification requires sufficient linkage disequilibrium (LD) between markers and a given QTL and large-scale genotyping.

Several factors affect the accuracy of genomic breeding values (GEBV) such as linkage disequilibrium (LD) between markers, size of training population and its relationship with target population, heritability of the trait, and the number of independent *loci* affecting the trait. Among these factors, the extent of LD can be highlighted since GS implicitly assumes a substantial LD between markers and QTLs, and also that, for each QTL, there is a marker in strong LD [8, 11]. Markers and QTLs should be in the same LD phase across breeds when carrying out GS using a multi-breed training population. The persistence of phase, which measures the genetic relationship between two populations, depends in part on the divergence time between populations and can be compared at many levels (between breeds, countries, or populations of the same breed and within the same country but for different generations [12]). The persistence of phase between breeds and the use of multi-breed training population for GS are important for populations with small number of genotyped and phenotyped animals as well as for production system that market crossbred animals.

The majority of pigs in the current Canadian breeding farms includes Duroc (DU), Landrace (LA) and Yorkshire (YO). Despite the knowledge of the LD pattern and persistence of phase in these breeds from other countries such as United States [13], Finland [14] and Denmark [15], to date, there is still a lack of information for Canadian animals. Furthermore, it is also important to evaluate these parameters in crossbred animals. As in many other countries, the Canadian pig industry consists of a three-level pyramidal structure and its success depends greatly on improvements achieved at the nucleus level, which are transferred down the pyramid to commercial operations. Nucleus breeders at the top work to genetically improve each breed using the most advanced selection methods. Multiplier herds then cross major breeds to produce hybrid breeding stock. Hybrids are then transferred to commercial operations where the final product, usually a three-way cross, is produced by more than one million commercial sows. For such systems, the breeding goal in purebred populations should be optimizing the performance of crossbred progeny [16]. Another important parameter to be evaluated is the genetic diversity of a population, as this is relevant to the sustainable use of genetic resources and continued long-term genetic improvement [17]. For instance, knowledge of the current effective population size, levels of inbreeding and of genetic diversity metrics in Canadian pig breeds can help geneticists to define better management strategies for the Canadian pig herds.

Thus, the objectives of this study were: 1) to investigate genetic diversity levels; 2) to estimate genome-wide extent of linkage disequilibrium; and, 3) to explore the persistence of phase between herds and breeds in three major Canadian purebred pig populations and one crossbred population to evaluate the possibility of a multi-herd and multi-breed training population for genomic prediction of breeding values.

## Methods

### Animals and genotypes

A total of 3,057 Duroc (DU), Landrace (LA), Yorkshire (YO), and crossbred Landrace × Yorkshire (F1) pigs (Table 1), born between 2001 and 2010 (DU), 1998 and 2010 (LA), 2000 and 2011 (YO), and 2008 and 2009 (F1), were included in this study. These animals were sampled from herds distributed across Canada, which are part of the Canadian Swine Improvement Program coordinated by the Canadian Centre for Swine Improvement (CCSI, https://www.ccsi.ca/).

**Table 1:** Number of genotyped animals in three purebred and one crossbred Canadian pig populations

Breed	Number of genotyped animals				
	H1	H2	H3	H4	Total
Duroc	403	215	141	307	1,066
Landrace	203	249	116	200	768
Yorkshire	359	221	85	446	1,111
Crossbred^a^	-	-	-	-	112

^a^Landrace × Yorkshire; H1, H2, H3 are closed herds and H4 consists of animals from 45 herds which share genetics among each other

Genotyped animals included key ancestors, parents, littermates, and performance tested animals with carcass and meat quality measures (tested at the Deschambault swine testing station located in Deschambault, Quebec, Canada). Animals were genotyped with the Illumina Porcine SNP60 BeadChip (Illumina, San Diego, CA) [18]. The SNP physical positions were obtained from the pig genome assembly 10.2 (Sscrofa10.2), (Martien Groenen, Wageningen University, data downloaded from the AnimalGenome.org data repository (http://www.animalgenome.org/repository/pig/) on 2013-March-01). A total of 62,163 SNPs were mapped to a genomic position, of which 55,396 SNPs were located on autosomal chromosomes and 1,550 SNPs were located on X chromosome; 5,217 SNPs did not have a known position. For genotyping quality control, the autosomal SNPs were filtered according to four criteria: SNP call rate ≥ 90%, minor allele frequency ≥ 0.05, *p*-value of χ^2^ test for Hardy-Weinberg equilibrium ≥ 10^−6^, and animal call rate ≥ 90%.

Possible misplaced SNPs were identified in three purebred populations (DU, LA, and YO), by means of a simple algorithm that considers the decay of LD across genomic distance and the frequency of unexpectedly large linkage disequilibrium of distantly located SNPs. For the three breeds, the plot of LD decay was analysed to assist in the identification of remaining SNPs with unexpected patterns of LD. In total 608 SNPs were identified as possible misplaced SNPs (Additional file 1). The pattern of LD before and after the exclusion of these 608 SNPs are shown in Additional files 2 and 3, respectively. Fernández et al. [19] also reported the occurrence of position error in the pig genome Assembly 10 in a crossbred pig population. These procedures were carried out because preliminary results of LD analysis showed unexpected decreasing patterns of *r* ^2^ (Additional file 2), indicating possible errors in the SNP positions.

### Genetic diversity metrics

The metrics used to estimate levels of within-breed genetic diversity and population history were:

1. **Heterozygosity**: Observed heterozygosity (H_O_) was calculated as the number of heterozygous loci divided by the total number of loci. The observed heterozygosity was then compared to expected heterozygosity (H_E_).
2. **Average minor allele frequency** (**MAF**): MAF is the observed frequency of the least common allele.
3. **Average pairwise genetic distance** (**D**): The average pairwise genetic distance separating individuals within each population was calculated using PLINK package [20]. Larger values indicate greater genetic distance among individuals within a population. The average proportion of alleles shared was calculated as: ${D}_{ST} = \frac{IBS2+0.5*IBS1}{N}$, where IBS1 and IBS2 are the number of loci which share either 1 or 2 alleles identical by state (IBS), respectively, and N is the number of loci tested. Genetic distance between all pair-wise combinations of individuals was calculated as: D = 1 - D_ST_.
4. **Inbreeding coefficients**: The following measures of inbreeding were calculated for each individual:
  - **Excess of homozygosity** (**F**_**EH**_): $\frac{1}{m}{\displaystyle {\sum}_{i=1}^m1 - \frac{c_i\ \left(2 - {c}_i\right)}{2{p}_i\left(1 - {p}_i\right)}}$, where *m* is the number of SNPs, *p*_*i*_ is the frequency of the first allele and *c* is genotype call (i.e. the number of copies of the first allele) [20].
  - **VanRaden** (**F**_**VR**_): The F_VR_ estimate was calculated following VanRaden [21] based on the additive variance of genotypes. F_VR_ was derived from: ${F}_{VR} = \frac{{\displaystyle {\sum}_{i=1}^m}{\left[{c}_i-E\left({c}_i\right)\right]}^2}{2{\displaystyle {\sum}_{i=1}^m}{p}_i\left(1-{p}_i\right)}-1=\frac{{\displaystyle {\sum}_{i=1}^m}{\left({c}_i-2{\hat{p}}_i\right)}^2}{2{\displaystyle {\sum}_{i=1}^m}{p}_i\left(1-{p}_i\right)} - 1$. This was equivalent to estimating an individual’s relationship to itself (diagonal of the SNP-derived genomic relationship matrix, GRM) [22].
  - **Runs of homozygosity** – **ROH** (**F**_**ROH**_): F_ROH_ was calculated as the sum of regions of the genome that consists of runs of homozygosity divided by the total genome length across all 18 autosomes [23] covered by SNPs. Runs of homozygosity were identified and characterized using PLINK [20]. The ROH were defined by a minimum of 40 homozygous SNPS. One heterozygous SNP and a maximum of two missing markers per ROH were permitted.
  - **Pedigree based inbreeding** (**F**_**PED**_): The pedigrees of animals were traced back to the founder populations and mean inbreeding coefficients per breed were calculated using the Colleau’s indirect method [24].

### Principal component analysis

To investigate the genomic composition of the population, the principal components were derived from the genomic relationship matrix (**G**, [21]) calculated using all the genotyped animals and SNPs (after QC process). Principal components were calculated using the *prcomp* function of R package [25].

### Effective population size

The effective population size (N_e_) in each generation was calculated based on the average linkage disequilibrium (*r* ^2^, described in the next section) of different distances, assuming a model without mutation, using the formula described by Sved [26]: $E\left({r}^2\right)=\frac{1}{1+4{N}_ec}$, in which *c* is the distance in Morgans between the SNPs and *T* is equal to 1/2c and represents the age of N_e_ [27]. The N_e_ was estimated for different generations using the average of *c* (assuming *1 cM* = *1 Mbp*) and *r* ^2^ at every 0.10 (±0.05) Mbp for distances between 0.05 Mbp and 10 Mbp and 0.5 (±0.05) Mbp for distances between 10 and 20 Mbp.

### Extent of linkage disequilibrium

Linkage disequilibrium (LD) was determined using the squared correlation between alleles of two SNPs (*r* ^2^) and calculated for each pair of *loci* on each chromosome according to Hill and Robertson [28] and Lynch and Walsh [29]. The equation is represented as follows: ${r}^2=\frac{D^2}{f(A)\times f(a)\times f(B)\times f(b)}$ in which, $D=\frac{N}{N-1}\left[\frac{4{N}_{AABB}+2\left({N}_{AABb}+{N}_{AaBB}\right)+{N}_{AaBb}}{2N}-2\times f(A)\times f(B)\right],$ where, f (A), f (a), f (B) and f (b) are the frequencies of alleles A, a, B and b, respectively and N is the total number of individuals.

To evaluate the LD pattern along chromosomes, the data was sorted into groups based on pair-wise marker distances, defined every 0.01 Mbp until 5 Mbp, and the average of each group was then estimated. Analysis were performed using the software SNPPLD (Dr. Mehdi Sargolzaei, University of Guelph, Canada).

### Persistence of phase across breeds and herds

The persistence of phase was evaluated across breeds (DU, LA, YO, and F1) and across herds (H1, H2, H3, and H4). Crossbred animals were all from the same herd; DU, LA, and YO animals were from three closed herds (H1, H2, and H3), and one combined group of 45 pig breeding herds (H4). The number of animals by herd and breed is presented in Table 1. The persistence of phase was measured as the Pearson correlation between the average means of linkage phase in different distances. The persistence of phase was determined by taking the square root of *r* ^2^ value and assigning the appropriate negative or positive sign based on the calculated *D* value.

## Results

### Animals and genotype data

Purebred animals from three breeds, namely Duroc, Landrace, and Yorkshire, and one crossbred population (Landrace × Yorkshire, F1) were genotyped using the Porcine 60 K Illumina BeadChip panel, which contains 62,163 SNPs. The number of animals genotyped in each population is described in Table 1 and the number of SNPs excluded due to the quality criteria threshold applied and the number of remaining SNPs is shown in Table 2.

**Table 2:** Number of autosomal SNPs excluded during the quality control procedure of autosomal SNPs

Breed	Excluded SNPs			Remaining SNPs^b^
	MAF < 0.05	SNP CR < 0.90	HWE *p*-value < 0.00001	
Duroc	16,815	2,849	4,503	34,927
Landrace	10,136	2,849	1,251	42,164
Yorkshire	10,260	2,837	1,905	42,121
Crossbred^a^	10,934	2,593	1,756	42,325

*MAF* minor allele frequency, *CR* call rate, *HWE* χ^2^-test for Hardy-Weinberg equilibrium, ^a^: Landrace x Yorkshire, ^b^: after exclusion of 608 possible misplaced SNPs

The average distance between adjacent SNPs, after quality control and exclusion of possible misplaced SNPs, was higher for DU (0.07 Mbp), than for LA, YO, and F1 (0.06 Mbp) populations. The largest distance between adjacent SNPs was observed on chromosome 3 for DU (4.87 Mbp) and chromosome 2 for YO (2.82 Mbp), F1 (2.82 Mbp), and LA (2.62 Mbp) populations.

### Population structure and genetic diversity

**Fig. 1:**
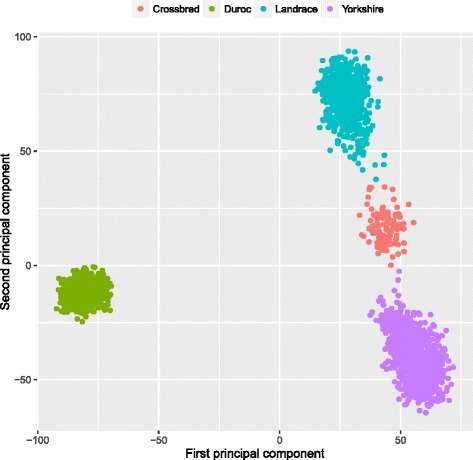
Principal component decomposition of the genomic relationship matrix colored by breed (PC1: 6.36% and PC2: 4.69%)

The first two principal components clearly discriminate all the breeds and F1 animals included in this study by revealing four main clusters represented by Duroc, Landrace, Yorkshire and Crossbred (Landrace x Yorkshire, F1) (Fig. 1). The first two PCs explained 6.36% and 4.69% of the total variation. As expected, F1 was situated between Landrace and Yorkshire. Landrace, Yorkshire and F1 are genetically more similar among themselves compared to Duroc.

**Fig. 2:**
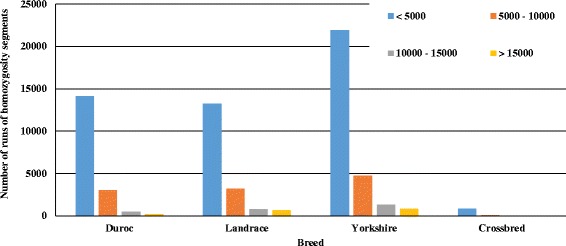
Number of runs of homozygosity segments in each length category for Canadian pig breeds

**Fig. 3:**
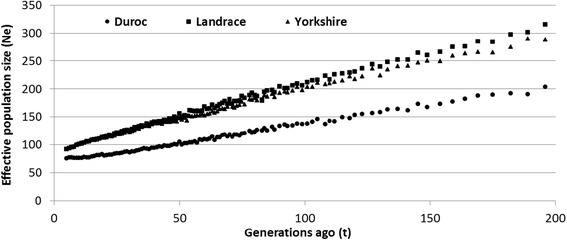
Estimates of effective population size (N_e_) for Canadian Duroc, Yorkshire and Landrace pig populations

Table 3 shows the genetic diversity metrics and a characterization of runs of homozygosity in the pig genome. Landrace and F1 displayed the highest levels of observed and expected heterozygosity. However, the differences among all the breeds were small. The average genetic distance between individuals was 0.30, 0.31, 0.30 and 0.28 within Duroc, Landrace, Yorkshire and Crossbred, respectively. The average MAF ± SD was 0.28 ± 0.13, 0.29 ± 0.13, 0.28 ± 0.13 and 0.29 ± 0.13 for Duroc, Landrace, Yorkshire and F1, respectively. There were differences between populations in terms of number and length of ROH (Fig. 2). Crossbred animals presented the lowest average number of ROH segments (NSEG, 8.25 ± 3.92) and Yorkshire presented the highest NSEG (25.88 ± 5.71). In general, Landrace and Yorkshire presented the highest number of ROH segments, which were larger in size and contained a greater number of SNPs per segment (Table 3). The inbreeding coefficients were similar among the purebred animals and lower for F1 animals, as expected (Table 3). Despite of the low to moderate inbreeding levels in the purebred animals, there were individuals with high inbreeding coefficients, indicating the need to account for inbreeding when planning matings. Table 4 shows the Pearson correlations among alternative inbreeding measures per population. For all purebred animals, F_PED_ presented a higher correlation with F_EH_, followed by F_ROH_ and F_VR_. The highest correlation (0.79) was observed between F_ROH_ and F_VR_ for crossbred animals. The effective population size in each generation is shown on Fig. 3. N_e_ at five generations ago was equal to 75 for DU and 92 for both LA and YO breeds, while 400 generations ago N_e_ was approximately 328 for DU, 515 for LA and 478 for YO.

**Table 3:** Genetic diversity, alternative inbreeding measures and characterization of runs of homozygosity in Canadian pig breeds

Parameter		Breed			
		Duroc	Landrace	Yorkshire	Crossbred
H_E_ ± SD		0.37 ± 0.12	0.38 ± 0.12	0.37 ± 0.12	0.37 ± 0.11
H_O_ ± SD		0.36 ± 0.12	0.37 ± 0.12	0.36 ± 0.11	0.42 ± 0.14
D_ST_		0.30	0.31	0.30	0.28
MAF ± SD		0.28 ± 0.13	0.29 ± 0.13	0.28 ± 0.13	0.29 ± 0.13
Inbreeding coefficients					
F_PED_	mean ± SD	0.07 ± 0.03	0.04 ± 0.04	0.05 ± 0.04	0.00 ± 0.00
	min	0.00	0.00	0.00	0.00
	max	0.27	0.33	0.29	0.00
F_ROH_	mean ± SD	0.03 ± 0.01	0.05 ± 0.02	0.05 ± 0.02	0.01 ± 0.01
	min	0.01	0.01	0.00	0.00
	max	0.06	0.14	0.18	0.07
F_EH_	mean ± SD	0.04 ± 0.06	0.03 ± 0.06	0.03 ± 0.06	−0.10 ± 0.04
	min	−0.23	−0.17	−0.31	−0.17
	max	0.37	0.33	0.32	0.16
F_VR_	mean ± SD	0.04 ± 0.08	0.03 ± 0.07	0.03 ± 0.08	−0.11 ± 0.09
	min	−0.12	−0.13	−0.12	−0.19
	max	0.38	0.32	0.33	0.03
Runs of homozygosity					
NSEG	mean ± SD	16.72 ± 3.66	23.19 ± 6.80	25.88 ± 5.71	8.25 ± 3.92
	min	2	0	0	2
	max	28	45	45	38
KB	mean ± SD	67,468 ± 18,889	112,729 ± 46,956	119,948 ± 42,314	26,519 ± 17,652
	min	5,393	0	0	5,050
	max	138,427	353,376	445,224	178,955
KB_AVG_	mean ± SD	4,033 ± 745	4,808 ± 1,519	4,584 ± 1,269	3,204 ± 1,047
	min	2,573	0	0	2,262
	max	9,345	13,110	13,492	12,194
NSNP		91.24	113.80	108.60	76.38
Density		43.68	41.67	41.59	41.72

*F*_*EH*_, *F*_*VR*_, *F*_*ROH*_ and *F*_*PED*_ inbreeding coefficients based on excess of homozygosity, VanRaden, runs of homozygosity and pedigree, respectively, *NSEG* Average number of segments for the individual declared homozygous, *KB* Average of total number of kb contained within homozygous segments, *KB*_*AVER*_ Average size of homozygous segments, *NSNP* average number of SNPs in run, *min* minimum, *max* maximum; *SD* standard deviation

**Table 4:** Pearson correlations among alternative inbreeding coefficients

	Duroc			Landrace			Yorkshire			Crossbred		
	F_ROH_	F_EH_	F_VR_	F_ROH_	F_EH_	F_VR_	F_ROH_	F_EH_	F_VR_	F_ROH_	F_EH_	F_VR_
F_EH_	0.41			0.72			0.69			0.64		
F_VR_	0.17	0.29		0.48	0.49		0.18	0.06		0.79	0.51	
F_PED_	0.31	0.65	0.31	0.32	0.40	0.24	0.53	0.55	0.20	0.00	0.00	0.00

*F*_*EH*_, *F*_*VR*_, *F*_*ROH*_ *and F*_*PED*_ inbreeding coefficients based on excess of homozygosity, VanRaden, runs of homozygosity and pedigree, respectively

### Extent of linkage disequilibrium

**Fig. 4:**
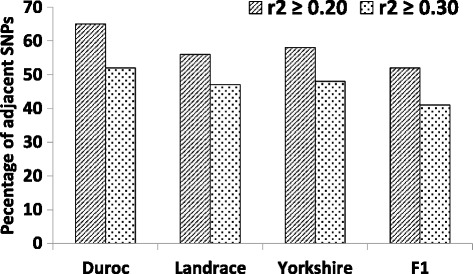
Percentage of adjacent SNPs with useful *r* ^2^ observed in four populations of Canadian pigs. Animals were genotyped for the Porcine 60 k Illumina BeadChip and Crossbred is Landrace × Yorkshire

The overall LD (*r* ^2^) across the genome between adjacent autosomal SNPs was 0.49, 0.38, 0.40 and 0.31 for DU, LA, YO and F1, respectively. The average *r* ^2^ in the autosomal chromosomes ranged from 0.39 to 0.59 for DU, 0.33 to 0.44 for LA, 0.34 to 0.45 for YO, and 0.25 to 0.39 for F1. The highest average LD was observed on chromosome 14 for DU, LA and F1 and on chromosome 13 for YO, while chromosome 10 showed the lowest average *r* ^2^ across all four populations. For all chromosomes, DU had the greatest LD followed by YO, LA and F1. The percentage of adjacent SNPs with *r* ^2^ ≥ 0.20 and *r* ^2^ ≥ 0.30 is shown on Fig. 4.

**Fig. 5:**
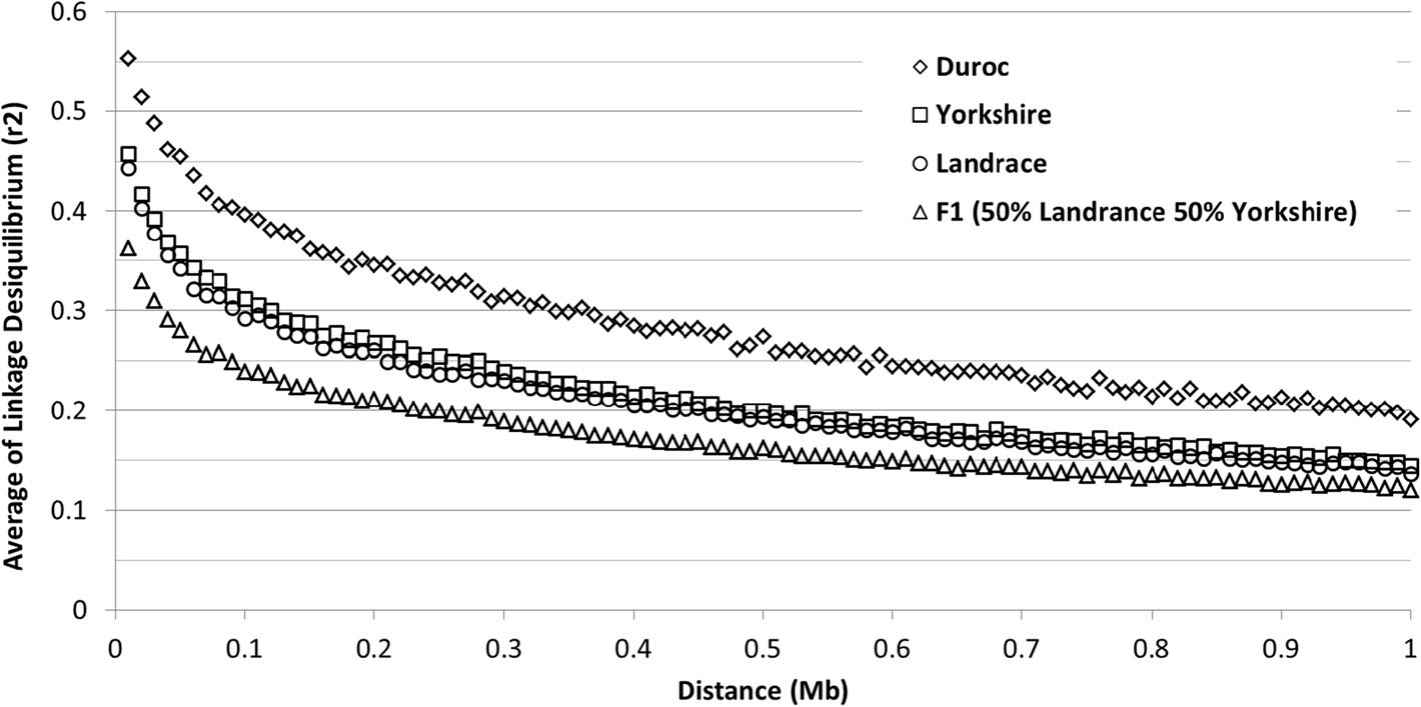
Average *r* ^2^ values at distances up to 1 Mbp for Canadian pigs. Linkage disequilibrium was estimated using information of the 60 k SNP panel on three purebred and one crossbred population

The decline of LD according to distance, for autosomal pair-wise SNPs up to 1 Mbp is shown in Fig. 5. The average *r* ^2^ between pair-wise SNPs followed the same pattern as adjacent SNPs: DU has a stronger *r* ^2^ at all distances, followed by YO, LA and F1. An average of *r* ^2^ ≥ 0.20 was observed at distances of 0.98 Mbp for DU, 0.50 Mbp for YO, 0.45 Mbp for LA, and 0.25 Mbp for F1. At 0.1 Mbp, the average *r* ^2^ between pair-wise SNPs for DU and YO populations was higher than 0.30, while for LA and F1 it was equal to 0.29 and 0.24, respectively.

The levels of LD at different distances are presented in Table 5. DU had the strongest LD, followed by YO, LA and F1. For distances up to 1 Mbp, a small difference (0.01) on average *r* ^2^ was observed between LA and YO. Similar levels of LD were observed for LA and YO at distances greater than 1 Mbp and for LA, YO and F1 at distances greater than 2.1 Mbp.

**Table 5:** Average *r* ^2^ values, estimated using the 60 k SNP panel, in four Canadian pig populations

Distance (Mbp)	Duroc	Landrace	Yorkshire	Crossbred^a^
0.00–0.01	0.61	0.51	0.52	0.41
0.01–0.05	0.49	0.38	0.40	0.31
0.05–0.10	0.42	0.31	0.33	0.26
0.10–0.50	0.32	0.23	0.24	0.19
0.50–1.00	0.23	0.16	0.17	0.14
1.00–2.00	0.16	0.11	0.12	0.10
2.00–3.00	0.11	0.08	0.08	0.08
3.00–4.00	0.08	0.06	0.06	0.07
4.00–5.00	0.07	0.05	0.05	0.06

^a^Landrace × Yorkshire

### Persistence of gametic phase across breeds and across herds

**Fig. 6:**
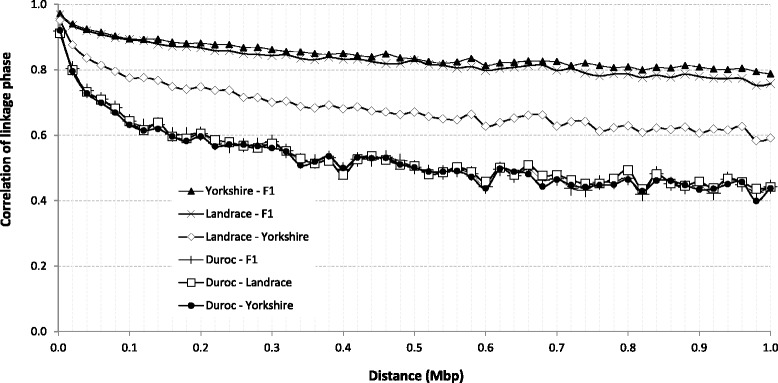
Persistence of gametic phase between four Canadian pig populations

**Fig. 7:**
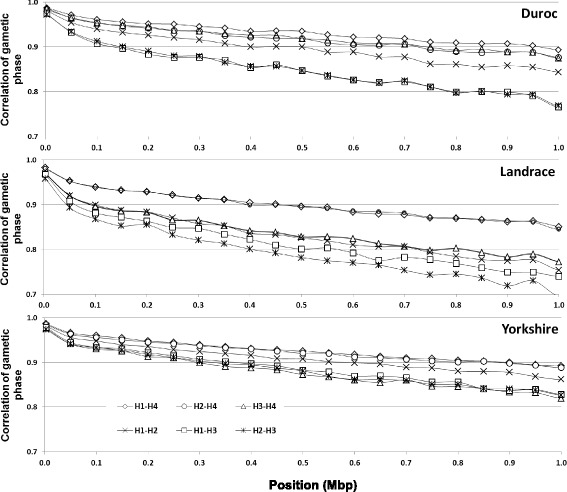
Persistence of gametic phase between four herds of three Canadian purebred pig populations. Points were plotted just every 0.05Mbp for better visualization. H1, H2 and H3 are closed herds and H4 includes animals from 45 different herds where genetics are exchanged among these herds

The persistence of gametic phase between two populations (breeds or herds) was evaluated using the Pearson correlation coefficient (r) using the gametic phase mean of two populations at different distances. Persistence of gametic phase across breeds is presented in Fig. 6 and across herds is presented in Fig. 7.

The highest correlation (*r* ≥ 0.90) was observed between F1 and the maternal breeds (LA and YO), at a distance up to 0.1 Mbp (Fig. 6). At the same classes of distances, LA presented *r* ≥ 0.80 with YO. A smaller value (*r* ≥ 0.68) was observed between DU and other breeds (LA, YO, and F1). The decay of *r* over the distances was more evident when comparing DU and maternal purebreds (YO or LA) than when both maternal breeds (LA versus YO) were compared.

Persistence of gametic phase across herds was calculated for purebred populations (DU, LA and YO) in order to evaluate whether the different selection processes applied to different herds generate genetic divergence between groups (Fig. 7). Each purebred population was found in three closed herds (H1, H2, and H3), and open group (H4), the latter including animals from 45 herds that exchange pig genetics among each other. The LA population showed more divergence between herds, with a rapidly decreasing correlation between groups, followed by DU and YO breeds. Except for the YO breed, the H3 group was less correlated with H1 and H2 than with H4 for all populations; the lowest correlation was found between H3 and H4 groups. In general, the open herd consisting of animals from numerous farms (H4) had the greatest correlation with the other (closed) herds.

## Discussion

### Animals and genetic diversity

The 60 K SNP panel, after the quality control and excluding possible misplaced SNPs, showed good coverage of the porcine genome with an average gap size equal to 0.07 Mbp for DU and 0.06 Mbp for LA, YO, and F1 populations. The average gap size and number of SNPs in this study (Table 2) was close to those reported by Badke et al. [13] for US pigs and Veroneze et al. [30] for 6 commercial pig lines.

The average genetic distance (D_ST_) between individuals was higher than previous studies reported in the literature such as Ai et al. [31] whom reported D_ST_ ranging from 0.11 ± 0.02 (Ganxi) to 0.23 ± 0.04 (Kele) within Chinese pigs and 0.24 (Duroc) to 0.29 (Large White) in Western breeds. The higher values of genetic distance observed in our study indicate a greater variability within the pig populations investigated. A greater genetic variability is beneficial for genetic selection purposes. The moderate MAF observed in these populations indicates the adequacy of the current SNP Chip for the genotyped breeds, as the majority of SNPs are informative and useful for genome-wide association studies and genomic prediction of breeding values.

In the present study, both PCA plots and persistence of gametic phase indicated a greater genetic similarity between LA and YO (and F1) and a more distant relationship with Duroc (Fig. 1, Fig. 6). As discussed in Wang et al. [15] the closer relationship between Landrace and Yorkshire is in agreement with their breeding history, as these two breeds were crossed around 1890 and the herdbook decided to keep them apart soon later.

The metric runs of homozygosity (ROH) can be used as an indicative of demographic history processes (e.g. bottlenecks, demographic expansion, effective population size) and levels of inbreeding in the population [32, 33]. Studies have shown that individuals with long ROH segments have greater inbreeding levels and FROH has also shown a good correlation with pedigree inbreeding coefficients [33, 34]. We assessed autozygosity as runs of homozygosity (ROH), and expected higher proportion of longer ROH in recently inbred populations. Landrace and Yorkshire presented a higher proportion of longer ROH segments compared to the other populations, suggesting higher levels of recent inbreeding in these breeds and thus lower individual genetic diversity. A characterization of ROH in pigs has also been previously reported by Herrero-Medrano et al. [35] for pig populations from the Iberian Peninsula. The authors reported a mean of the total number of ROH per population between 24 and 34, which are slightly higher than the values reported in the present study, however, consistent with the breeds’ history. The low number of long ROH observed in the F1 animals reflects the effects of crossbreeding on breaking down the long ROH segments. As discussed in Herrero-Medrano et al. [35], the assessment of ROH at the individual level has also practical implications, as animals displaying high levels of ROH, for instance, could be excluded or given lower priority for breeding purposes in endangered populations.

Alternative genomic inbreeding estimates were evaluated and compared with pedigree-based inbreeding. In general, genomic markers traced the same trends in inbreeding as pedigree. For Duroc, average F_PED_ was higher than the genomic inbreeding coefficients. The majority of inbreeding metrics was moderately correlated among themselves. The low correlation observed for F_EH_ and F_VR_ for the Yorkshire breed is probably due to differences in the allele frequencies calculations in both methods. Interestingly, the correlation between F_VR_and F_ROH_ in F1 was the highest correlation (0.79). F_VR_ requires the calculation of allele frequency in the base population and as F1 animals are crosses between Landrace and Yorkshire, we suspect that their allele frequencies are more similar to the allele frequencies in the base population (pure breeds). Despite the low to moderate levels of inbreeding in these populations, there were animals with high inbreeding coefficients and therefore this information should be accounted in the mating decisions. Furthermore, we reported moderate correlations between F_ROH_ and F_PED_, indicating that the information on ROH could also contribute in the selection of animals for mating in order to reduce inbreeding.

The N_e_ values calculated in the present study are in agreement with values reported by Uimari and Tapio [14] for Finnish Landrace (N_e_ = 91) and Finnish Yorkshire (N_e_ = 61) populations, estimated at five generations ago using pedigree information. Welsh et al. [36] studied US pigs and reported an N_e_ at 17 generations ago equal to 100 for DU and YO breeds, whereas the N_e_ for LA was below 100. These results were similar to our findings; the calculated N_e_ was approximately 81 for DU and 110 for LA and YO breeds at 17 generations ago (Fig. 3).

Genomic data has also been used to investigate older genetic events in pig populations, such as the study reported by Groenen et al. [37], where the authors reported evidences of genetic events including bottlenecks, population expansion and admixture between wild and domestic pig breeds [38–40]. Our results show that N_e_ has suffered a progressive decline through time in these populations and was less than 100 a few generations ago. Meuwissen [11] recommended an effective population size of 100 in order to maintain the genetic diversity of a population. Our findings are in accordance with Melka and Schenkel [41], who pointed out to the need of conservation strategies for Canadian pigs, especially for the DU breed. The N_e_ estimates were also used to calculate the number of markers needed to achieve accurate GEBV and it indicates that an accurate GEBV within breed can be expected using a panel containing approximately 30,000 SNPs (10*N_e_*L, [2]).

### Extent of linkage disequilibrium

The average LD between adjacent SNPs observed for purebred Canadian pigs (0.49 for DU, 0.40 for YO, and 0.38 for LA) as well as the decay of LD across distances (Fig. 5) were similar to the results reported by Badke et al. [13] for US pigs. The authors reported average *r* ^2^ of adjacent SNPs equal to 0.46 for DU, 0.39 for YO and 0.36 for LA breeds. The results regarding the average *r* ^2^ between adjacent SNPs and the extent of LD across distances reported by Veroneze et al. [30] for 6 commercial pig lines were also similar to our study.

Canadian pigs showed stronger LD than US pigs [13] for pair-wise SNPs at short distances (<50 Kb). Badke et al. [13] reported an average *r* ^2^, at short distances, lower than 0.40 for the Duroc breed and lower than 0.30 for LA and YO breeds. Our results showed an average *r* ^2^ greater than 0.50 for DU, LA, and YO breeds, and greater than 0.40 for F1 pigs. These differences may be attributed to the population structure of each breed, selection or sample size. Badke et al. [13] analyzed less than 100 animals for each breed, while the current study included more than 700 animals per breed. Wang et al. [15] reported *r* ^2^ values of 0.55, 0.50 and 0.50 for Danish Duroc, Landrace and Yorkshire. Park et al. [42] reported an *r* ^2^ of 0.48 for Korean Landrace. Veroneze et al. [43] reported *r* ^2^ values ranging from 0.46 to 0.55 at distances of 0 to 50 Kb.

Similar *r* ^2^ estimates were observed between Canadian, American [13] and Finnish [14] pig populations. According to Meuwissen et al. [11], an accuracy up to 85% can be achieved for genomic breeding values in dairy cattle when *r* ^2^ estimates are greater than 0.20 between adjacent SNP. Considering *r* ^2^ greater than 0.20 as a useful LD level, we observed that the studied Canadian pig populations had useful average LD between more than 50% of the adjacent SNPs (Fig. 4) and between pair-wise SNPs located up to the distance of 0.98 Mbp for DU, 0.50 Mbp for YO, 0.45 Mbp for LA, and 0.25 Mbp for F1 populations (Fig. 5). The level of LD for the crossbred line was lower than the LD level for purebred pigs (Fig. 5 and Table 5). However, these LD values are still greater than what has been observed in North American dairy cattle [44] indicating that genomic selection might be applicable for pig breeds, including crossbreds, considering that other requirements (such as proper training population and good phenotypic observations) are met.

### Persistence of gametic phase across breeds and across herds

Persistence of gametic phase can be used to investigate the history and relatedness of breeds within a specie as well as on reliability of across population GWAS and GEBV prediction [12]. High positive values are a result of equal phase in both breeds being contrasted. The persistence or correlation of gametic phase between maternal breeds (LA vs. YO, F1 vs. LA, and F1 vs. YO) was higher than the correlations between the paternal and maternal breed populations (DU vs. LA, DU vs. YO, and DU vs. F1, Fig. 6). These results are in agreement with previous results that reported higher correlation between LA and YO when compared to DU with either LA or YO breeds, for Canadian [45] and US pigs [13]. For distances up to 0.01 Mbp, the correlation of gametic phase between LA and YO (0.93), DU and LA (0.89), and DU and YO (0.89) breeds are in agreement with the values reported for US pigs [13]. When the distance between adjacent SNPs is increased up to 0.05 Mbp, the persistence of gametic phase decreased to 0.82 (LA vs. YO), 0.71 (DU vs. LA), and 0.72 (DU vs. YO), which is equal to the values reported for Canadian pigs [45] and slightly lower than for US pigs [13].

The correlation of gametic phase between Canadian pig breeds (Fig. 6) were above 0.80 for distances up to 1.07 (F1 with YO), 0.81 (F1 with LA), 0.08 (LA with YO), and 0.02 (DU vs. other populations) Mbp. Comparing these results with the results from cattle simulation study [46], we can expect favourable gain in genomic prediction reliability when combining F1 with either LA or YO breeds in a training population.

In cattle, De Roos et al. [46] evaluated the effect of combining multiple populations on the reliability of genomic predictions and concluded that the benefits of combining populations in a training set were higher under the following conditions: populations diverged only few generations ago, high marker density, or low heritability. These authors conducted simulation study and considered populations that have diverged for 6, 30, and 300 generations ago, which showed a correlation of phase greater than 0.8 for distances up to 0.45, 0.05 and 0.01 Mbp.

The presented persistence of gametic phase of LA with YO was lower than the correlation observed for populations that diverged six generations ago, but higher than those that diverged 30 generations ago. Therefore, results of the present study suggest that the use of LA and YO in the same training population may provide gain in the accuracy of GEBV and that it should be further investigated. DU had lower correlation of linkage phase with other breeds than the correlation observed between the simulated cattle populations that diverged 30 generations ago [46], which indicates that a higher density panel may be needed to achieve gains in genomic predictions reliability when combining the DU breed with any other population in a training population.

Erbe et al. [47] showed that in dairy cattle, an increase in the panel density did not generate satisfactory gains in accuracy for multi-breed genomic selection evaluations. The authors suggested that, in addition to the correlation of linkage phase, the percentage of QTL segregating in both breeds and the relationship between animals of different breeds may also strongly affect the gain in accuracy when using a multi-breed training population. Studies involving an across breed training population for pigs are still justified because the decrease in LD and correlation of linkage phase across Canadian pigs populations are different from those obtained in dairy cattle [12]. Our study and the results obtained in US pigs populations [14] showed that LD is extended for longer distances (Fig. 5) in pigs when compared to cattle, as well as the persistence of gametic phase across breeds (Fig. 6), especially for breeds with similar purposes in production (i.e. LA and YO breeds used as maternal lines).

When comparing the correlations obtained in this study with those reported for dairy cattle [12], lower values were found between Canadian herds than between US and Canadian Holstein (~0.90, for distances up to 10 Mbp) [44]. The small correlation between Canadian pig herds may be due to the different emphasis on selection process in each herd and a lower relationship between closed herds. These lower correlations between Canadian pig herds may indicate the need to have genotyped and phenotyped animals prevenient from all the herds involved in the genomic evaluations program.

## Conclusions

The 60 K SNP panel allows good coverage of the pig genome for Canadian Duroc, Landrace, Yorkshire, and F1 populations. Better coverage of the pig genome can be achieved with improvements on the *Sus Scrofa* genome map. Similar levels of genetic diversity were observed among all breed groups. Despite the low to moderate levels of inbreeding in these populations, there were animals with high inbreeding coefficients and therefore this information should be taken into account in the mating decisions. Effective population size has suffered a progressive decline through time, and it was less than 100 a few generations ago, indicating a need for management strategies to avoid reduction in genetic diversity. The analysis of runs of homozygosity also gave us insights about the populations’ demographic history.

The estimated average *r* ^2^ for the three Canadian pig breeds indicates that accurate genomic selection can potentially be implemented within breeds with the current 60 K SNP panel. A representative training population from all herds is essential due to the low/moderate persistence of gametic phase among them. The SNP panel used in our study may be suitable for multi-breed genomic evaluation involving F1, Landrace, and Yorkshire populations owing to higher phase consistency between these populations. The lower correlation of phase observed between Duroc and the other breeds indicates that a denser panel may be required for Duroc to be included in across-breed evaluations.

## Additional files

Additional file 1: List of possible misplaced SNP. Containing the names of the 608 SNPs identified as possible misplaced SNPs. (XLSX 18 kb)
Additional file 2: Pattern of linkage disequilibrium by chromosome (Chr) for Canadian pigs, before the exclusion of possible misplaced SNPs. Containing the pattern of linkage disequilibrium decay across distances, calculated using the *Sus scrofa* 10.2 assembly. (DOCX 672 kb)
Additional file 3: Pattern of linkage disequilibrium by chromosome (Chr) for Canadian pigs, after the exclusion of possible misplaced SNPs. Containing the pattern of decay of linkage disequilibrium across distances, after the exclusion of 608 possible misplaced SNPs and using the *Sus scrofa* 10.2 assembly. (DOCX 512 kb)

## Abbreviations

CR: Call rate
DU: Duroc
F1: Crossbred, Landrace × Yorkshire
GEBV: Genomic estimated breeding value
GS: Genomic selection
HWE: Hardy-Weinberg equilibrium
LA: Landrace
LD: Linkage disequilibrium
MAF: Minor allele frequency
Mbp: Mega base pairs
N_e_: Effective population size
QTL: Quantitative trait *locus*
R: Correlation of gametic phase
*r*^2^: Linkage disequilibrium
SNP: Single nucleotide polymorphism
YO: Yorkshire
