# Supplementary figures and images for: Genetic diversity, extent of linkage disequilibrium and persistence of gametic phase in Canadian pigs
Source: BMC Genet. 2017 Jan 21;18:6. doi: 10.1186/s12863-017-0473-y (PMC5251314; doi:10.1186/s12863-017-0473-y)

**Duroc**


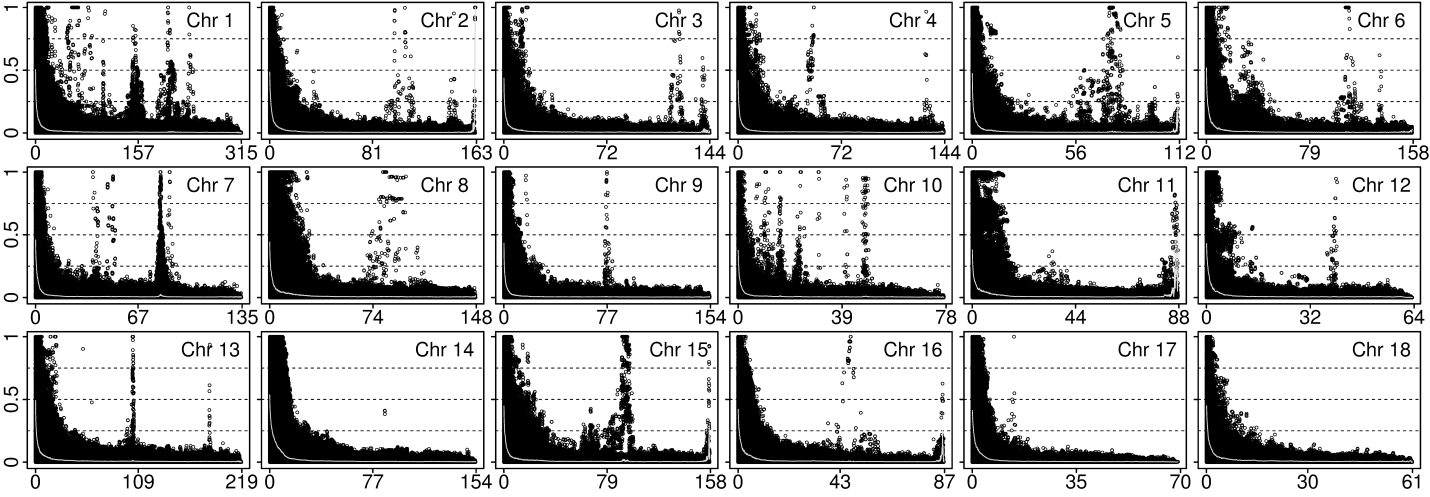


**Landrace**


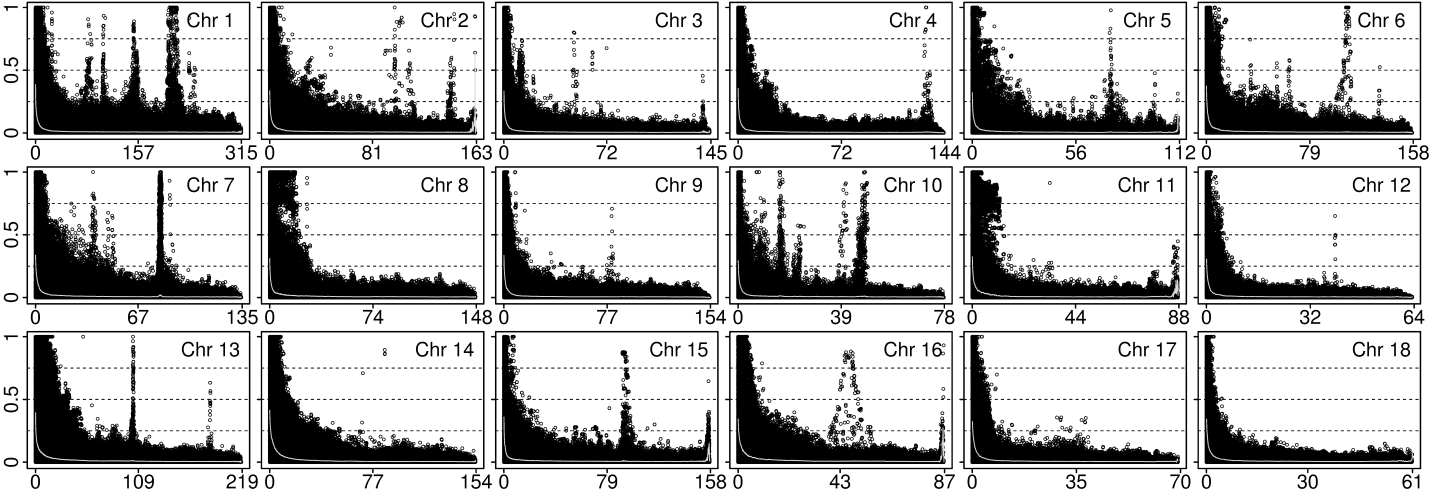


**Linkage Disequilibrium (r2)**

**Yorkshire**


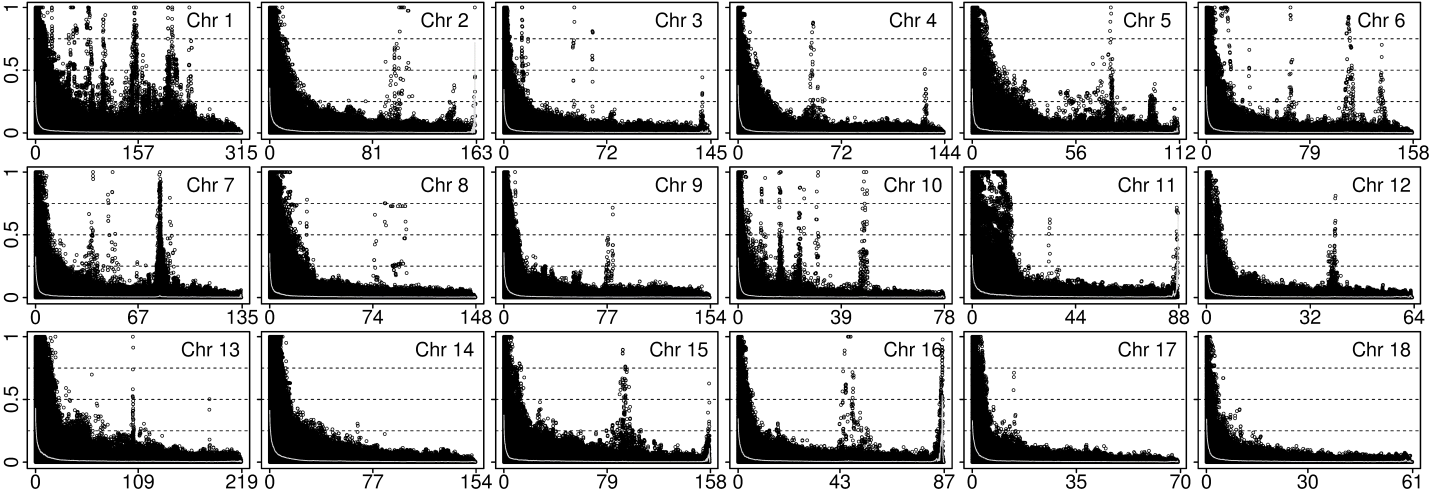


**Distance between SNPs (Mb)**

Supplement: Additional file 2: — Pattern of linkage disequilibrium by chromosome (Chr) for Canadian pigs, before the exclusion of possible misplaced SNPs. Containing the pattern of linkage disequilibrium decay across distances, calculated using the Sus scrofa 10.2 assembly. (DOCX 672 kb) [file 12863_2017_473_MOESM2_ESM.docx]

**Duroc**


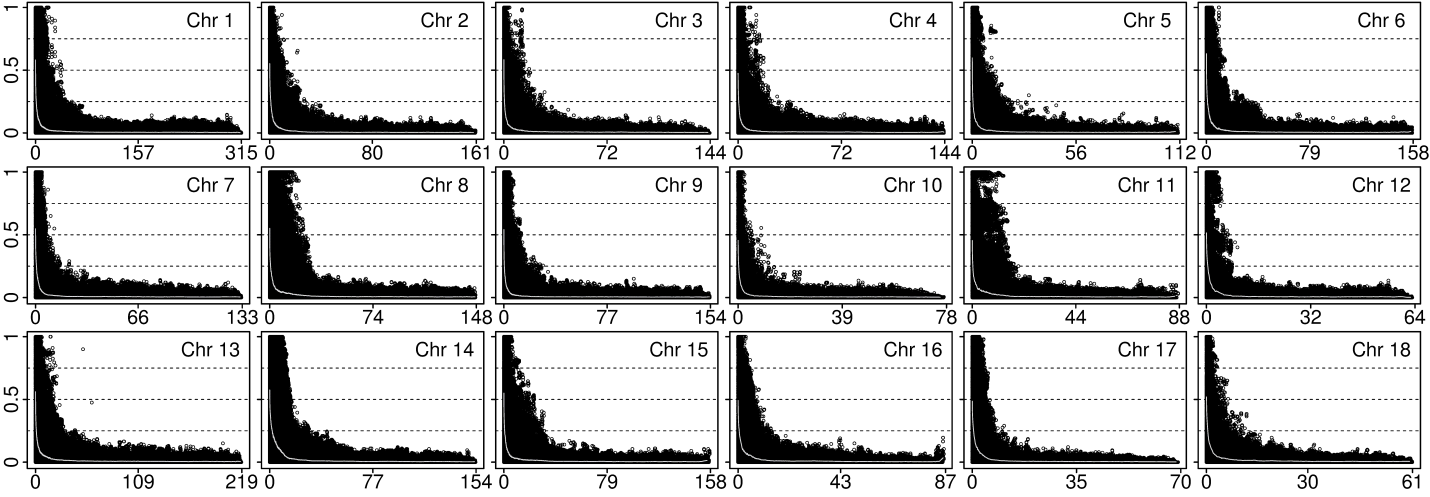


**Landrace**


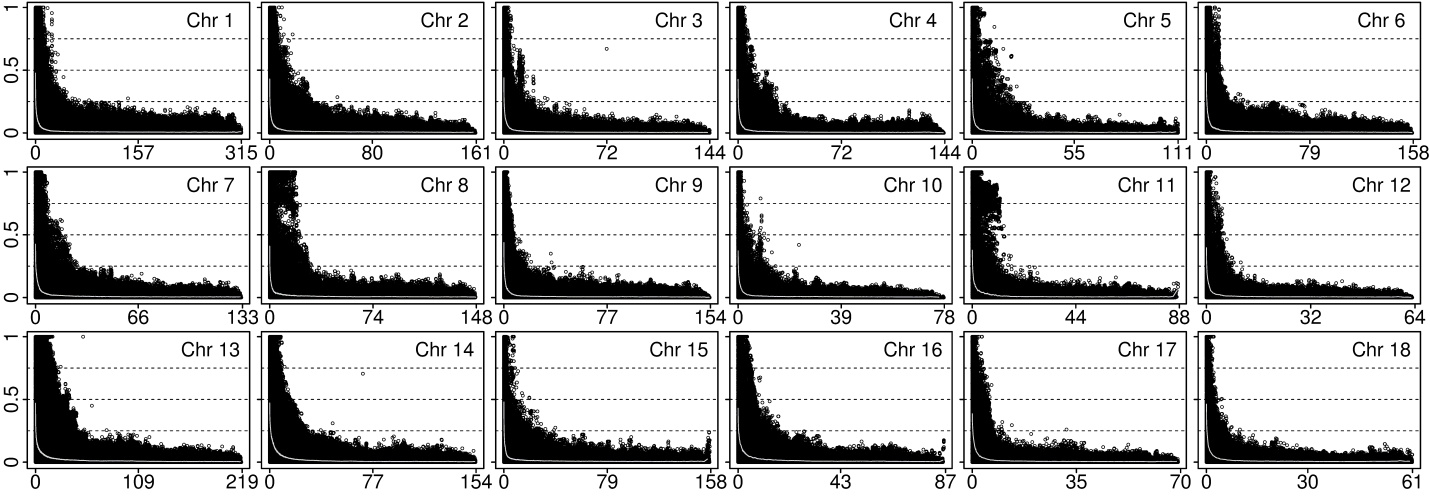


**Linkage Disequilibrium (r2)**

**Yorkshire**


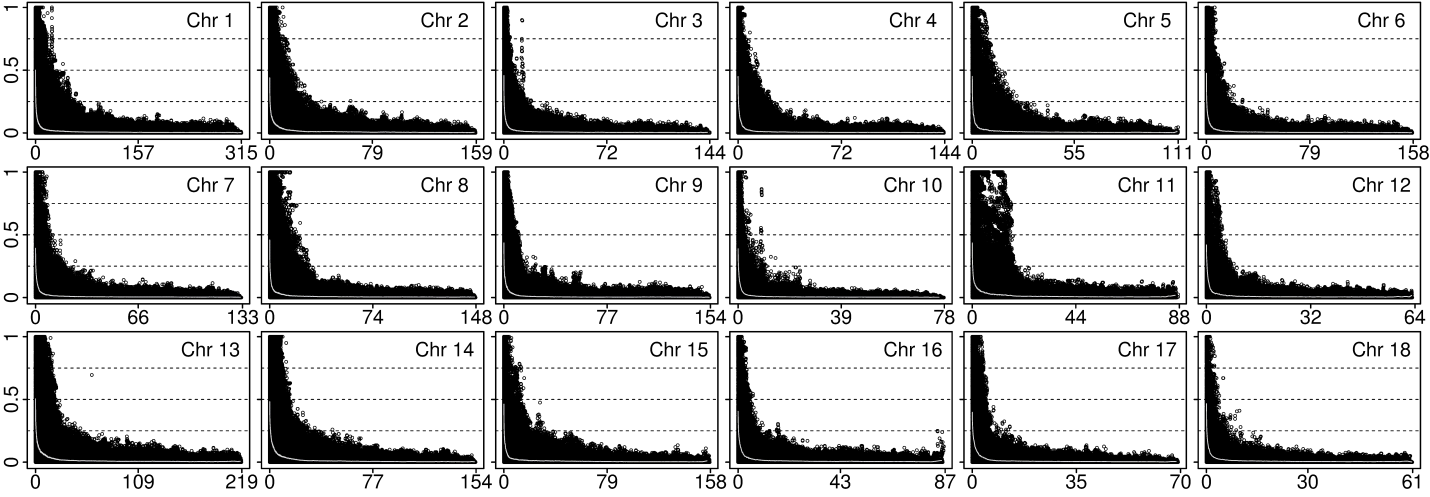


**Distance between SNPs (Mb)**

Supplement: Additional file 3: — Pattern of linkage disequilibrium by chromosome (Chr) for Canadian pigs, after the exclusion of possible misplaced SNPs. Containing the pattern of decay of linkage disequilibrium across distances, after the exclusion of 608 possible misplaced SNPs and using the Sus scrofa 10.2 assembly. (DOCX 512 kb) [file 12863_2017_473_MOESM3_ESM.docx]
